# Effects of perceived instructional support and self-regulated learning support on students’ learning behavior in an online learning environment

**DOI:** 10.1371/journal.pone.0332296

**Published:** 2025-09-17

**Authors:** Libor Juhaňák, Karla Brücknerová, Jakub Jarina, Jiří Zounek, Vojtěch Juřík

**Affiliations:** 1 Department of Educational Sciences, Faculty of Arts, Masaryk University, Czech Republic; 2 Centre for Information Technologies, Faculty of Arts, Masaryk University, Czech Republic; 3 Department of Psychology, Faculty of Arts, Masaryk University, Czech Republic; 4 Institute of Computer Aided Engineering and Computer Science, Faculty of Civil Engineering, Brno University of Technology, Brno, Czech Republic; University of Cambridge, UNITED KINGDOM OF GREAT BRITAIN AND NORTHERN IRELAND

## Abstract

Learning Analytics (LA) has advanced significantly in recent years; however, its findings often suffer from limited generalizability and transferability due to reliance on data from a small number of courses. Course design variability is a critical factor influencing students’ learning behavior in online learning environments (OLEs). This study examines how differences in instructional design, specifically in self-regulated learning (SRL) support, predict the intensity and regularity of students’ learning behavior in OLEs. Using qualitative content analysis, we developed the SRL-S coding scheme to systematically assess the extent to which course design supports specific SRL processes. The coding scheme was validated by three independent researchers and applied to 76 courses. Multilevel modeling analysis confirmed substantial variability in student learning behavior across courses. Higher SRL support was associated with more frequent course visits (β = .46, p < .001), greater regularity of visits (β = −.31, p < .001), and increased total time spent in a course (β = .45, p < .001). In contrast, perceived instructional support from the teacher significantly influenced total time spent (β = .12, p = .004) but not the visit frequency (p = .324) or regularity (p = .951). Moreover, SRL support significantly predicted perceived instructional support (β = .17, p = .002). Mediation analysis revealed a small but significant indirect effect of SRL support on total time spent (β = .021, p = .012), while no mediation was found for visit frequency (p = .360) or regularity (p = .836). These findings highlight the pivotal role of structured SRL support in shaping student engagement. Our approach contextualizes LA indicators by accounting for course design differences and suggests that the SRL-S coding scheme could serve as a research-based guideline for enhancing SRL support in online courses.

## Introduction

Learning Analytics (LA) has experienced remarkable growth in recent years, particularly in the context of online learning environments (OLEs) [1–3]. Over time, numerous metrics and indicators have been proposed to predict students’ learning outcomes or to enhance their learning experiences by monitoring these indicators [4]. Given the continuous evolution of OLEs, systematic research is essential to identify the fundamental mechanisms and design features that demonstrably contribute to learning effectiveness. Self-regulated learning (SRL) plays an increasingly considerable role in OLEs due to their unique organizational structures. SRL can profoundly influence students’ learning behaviors and outcomes, making it a key factor in the effectiveness of online education [5–8]. The instructional design of OLEs, particularly in terms of how it supports SRL, is expected to shape the intensity and regularity of students’ engagement with learning materials and, as such, should be intensively studied [9].

However, existing research in this area often struggles with issues of generalizability and transferability. Many studies rely on data from a limited number of courses [10–12], making it challenging to derive overarching conclusions and practical recommendations for course design. However, the course design, represented by considerable variability among various OLEs, is considered one of the most critical factors influencing students’ activity in OLEs—and, ultimately, their learning outcomes. Joksimović et al. [13] found that course-level differences can matter more than individual student differences in predicting success, while Gašević, Dawson, Rogers, & Gasevic [11] showed that LMS activity–outcome relationships are strongly moderated by course design, and ignoring this can bias predictive models. Despite this, few studies evaluate results across contexts and domains, even though this is critical for understanding student success [14]

Previous research suggests that differences in instructional approaches, structural organization, and support for SRL can significantly impact how students engage with their learning [11,13]. The present study aims to examine variations in course design, specifically in terms of their support for SRL, and to explore how these differences influence the intensity and regularity of students’ learning behavior in OLEs. This research provides valuable insights into optimizing course design to foster self-regulated learning and improve learning outcomes in online education by addressing these aspects.

### Learning analytics and measuring of student learning behavior

Learning Analytics (LA) has emerged as a powerful tool for examining and optimizing learning processes by analyzing student interactions within digital platforms [15,16]. Predictive modelling of academic success in blended learning, in particular, is considered a promising area, especially focusing on instructional conditions [17]. Commonly used LA indicators include frequency of logins, time spent on learning tasks, interaction with study materials, and participation in discussions [18]. The field of LA has grown significantly; however, its applicability has often been limited due to issues related to the generalizability and transferability of findings [11,13,19]. Given the variability in course design, it is essential to contextualize LA indicators to understand their implications for student learning behavior better [20].

Previous research indicates that differences in course structures contribute significantly to students’ engagement levels, learning persistence, and overall performance [21]. Consequently, an effective framework that captures the instructional design features influencing SRL is necessary for deriving actionable insights from LA data. Prior research has highlighted that instructional designs in OLEs exhibit significant variability, affecting the extent to which students engage in SRL [22]. Factors such as the presence of explicit goal-setting tasks, self-assessment activities, and reflective prompts contribute to the degree of SRL support within a given course [23].

### Instructional support in online learning environments

Instructional support in OLEs comprises various elements, including structured learning materials, scaffolding mechanisms, timely feedback, and interactive activities designed to enhance student engagement [24,25]. Research suggests that well-designed instructional support can improve students’ self-regulation, enhancing their ability to effectively manage learning tasks [26]. Studies indicate that different instructional support structures, such as guided prompts, formative assessments, and collaborative learning opportunities, influence the extent to which students engage with course content and regulate their learning behaviors [27].

Building on the previous section, it is necessary to distinguish instructional support from instructional design. Instructional design involves the systematic planning, organization, and structuring of course content, activities, and resources to optimize learning outcomes [28]. It focuses on the pre-planned layout and sequencing of the course rather than real-time interactions. In contrast, instructional support involves the guidance, scaffolding, feedback, and other assistance given to students as they engage with the learning environment [29]. By clarifying this distinction, we differentiate between the structural design of the course and the ongoing support students receive while learning.

While perceived teacher support is often considered a critical factor in student engagement, research suggests its impact on learning behavior may be indirect [30]. Instead, SRL support embedded in course design serves as a more direct determinant of students’ self-regulation and engagement levels [31], although the specific mechanisms of SRL still provide mixed evidence (see, e.g., [32,33]). To systematically measure the degree to which course designs support SRL, it is necessary to consider the creation of a specific research framework engaging coding scheme, aiming to assess the variability in SRL support and link these differences to students’ learning behavior, including regularity and intensity of engagement [34,35].

### SRL framework guiding the study

To anchor our analysis, this study adopts Zimmerman’s **cyclical model of self-regulated learning** [36–38], which conceptualizes SRL as a dynamic process consisting of three interrelated phases: (1) the preparatory phase, where learners set goals and plan strategies; (2) the performance phase, where learners engage in task execution, monitoring, and control; and (3) the reflective phase, where learners evaluate their progress and outcomes. This cyclical structure has been widely applied in SRL research because it captures both the proactive and reactive aspects of self-regulation and it encompasses metacognitive, cognitive, motivational, and behavioral processes that enable learners to set goals, choose strategies, and adapt their learning to achieve success [39]. However, it also engages time management, monitoring, self-evaluation, and self-reflection [40]. This model also aligns well with the affordances of online learning environments, in which students often must self-direct their progress over extended periods of time. In OLEs, where students often engage with content asynchronously and autonomously, SRL plays a critical role in determining learning effectiveness [41–45]. Given the flexible yet demanding nature of OLEs, SRL-supportive instructional designs become essential to fostering meaningful student engagement and learning outcomes [46]

Building on Zimmerman’s framework, we also incorporate the **cognitive–affective distinction** in SRL processes [25,47]. The cognitive dimension refers to mental strategies such as planning, monitoring, and applying learning techniques, while the affective dimension refers to motivation, self-efficacy, interest, and emotional engagement. This dual framing is especially relevant in OLEs, where both the management of learning strategies and the regulation of motivation/emotion determine the persistence and regularity of engagement [1]. In SRL, cognitive and affective dimensions are considered predictors of learning outcomes [47]. Cognitive support targets thinking and strategy use [38], whereas affective support focuses on emotional engagement [25]. Distinguishing these helps understand their influence on students’ online learning behavior. At the same time, the emotional dimension of learning in OLE is especially considered understudied [1] and should be further studied.

This integrated model provides the theoretical foundation for the methodological design of our study in several ways. First, the coding scheme for analyzing instructional design features was structured around the three SRL phases and further differentiated by whether a feature primarily supported cognitive or affective processes. Second, the selection and interpretation of learning analytics indicators were guided by this model. Finally, our research questions build directly on this framework by examining not only overall SRL support in OLEs but also the distinct contributions of specific phases and dimensions to students’ learning behavior.

By grounding the study in this combined cyclical-phase and cognitive–affective model of SRL, we provide a coherent theoretical lens that links instructional design, students’ perceptions of instructional support, and observable learning behaviors within online learning environments.

### Present study

Despite substantial progress in understanding self-regulated learning (SRL) in online learning environments (OLEs), at least four persistent research gaps remain. First, most empirical studies deal with individual courses or a limited number of courses. This results in a narrowly defined context that significantly limits the generalizability of findings to diverse online courses and environments [10,12]. Second, research often focuses on student-level variables while inadequately exploring how differences in course instructional design shape learning behavior. This is despite the fact that course-level differences have been proven to play a crucial role in shaping students learning activity and their learning outcomes in OLEs [11,13,22]. Third, SRL is often examined as a unitary construct, with little attention given to the distinct roles of the cognitive and affective dimensions or the preparatory, performative, and reflective phases that constitute its cyclical nature [1,38,42]. Finally, little attention has been given to the mechanisms linking the instructional design of SRL support at the course level to students’ perceptions of instructional support and how these two factors together affect the intensity and regularity of students’ online learning behavior in courses.

This study directly addresses the aforementioned gaps by collecting and analyzing a multi-source dataset from 76 different courses. This dataset combines instructional design data aimed at supporting SRL, which was collected through a qualitative content analysis of the courses; student self-reports of perceived instructional support, which were collected through a questionnaire; and behavioral traces of students’ learning behavior, which were collected from an authentic online learning environment. Thus, this study aims to contribute new knowledge to SRL research in OLE contexts by investigating (1) the extent to which SRL-supportive design features of courses predict student online learning engagement, (2) how these features interact with students’ perception of instructional support, and (3) how cognitive vs. affective dimensions and SRL phases differentially shape learning behavior.

Using qualitative content analysis of selected courses, we developed a coding scheme that enabled us to measure the extent to which individual courses’ design supports particular SRL processes. Three independent researchers critically reviewed the coding scheme and then applied it to the analysis of 76 different courses. To gain insight into how students perceived the courses, participants of analyzed courses were asked to fill in the online questionnaire mapping (among other things) the instructional support from the teacher within the course. To capture students’ learning behavior, several indicators of learning behavior in the individual courses were extracted from OLE at the end of the semester.

Based on this multi-source dataset, our study aims to answer the following research questions:

- **RQ1**: To what extent do the perceived instructional support from the teacher and the self-regulated learning support in OLE predict the intensity and regularity of students’ online learning behavior in courses?
- **RQ2**: To what extent does the self-regulated learning support in OLE predict perceived instructional support from the teacher?
- **RQ3**: Does the perceived instructional support from the teacher serve as a mediator for the relationship between the self-regulated learning support in OLE and the intensity and regularity of students’ online learning behavior in courses?
- **RQ4**: To what extent does the support of individual SRL dimensions (cognitive vs. affective) predict the intensity and regularity of students’ online learning behavior in courses?
- **RQ5**: To what extent does the support of individual SRL phases (preparatory, performative, reflective) predict the intensity and regularity of students’ online learning behavior in courses?

**Fig 1:**
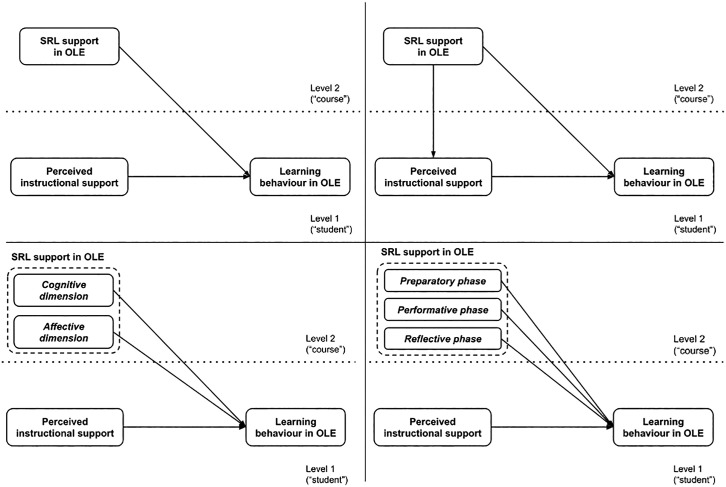
Conceptual models of the relationships considered in relation to the research questions.

The figure below (Fig 1) illustrates the models considered in relation to the research questions above. The conceptual model shown in the upper left part of the figure deals with research question 1 (RQ1). The upper right part relates to research questions 2 (RQ2) and 3 (RQ3). The lower left part shows the model addressing research question 4 (RQ4), and the lower right part addresses research question 5 (RQ5).

## Method

### Sample and procedure

A total of 882 students enrolled in 76 different courses held at the Faculty of Arts, Masaryk University, Brno, Czech Republic, were part of the analyses in this study. Only those students who completed the questionnaire and agreed that their OLE behavior data could be analyzed for this research were included in the sample from these courses. The study was conducted in accordance with relevant guidelines and regulations following the principles of Declaration of Helsinki, including approval from the ethics committee and written informed consent. Participants were briefed on the study’s purpose and asked to provide informed consent by selecting “I agree” in the online system before accessing the questionnaire. They were informed that they could refuse or withdraw from the study at any time without facing any consequences. The research project received approval from the Research Ethics Committee of Masaryk University (project identification number: EKV-2020–037). At the same time, only courses with responses from at least five different students were included in the sample (due to the planned multilevel analyses).

The mean age of the respondents was 21.98 years (Med = 21, SD = 4.25). As for gender, 76.87% of the respondents were female, 21.77% were male, and 1.02% of the students chose the option ‘other.’ This distribution is fairly consistent with the overall gender distribution of students at the Faculty of Arts. Both bachelor (77.55%) and non-follow-up master (21.77%) students were approached to participate in this research and complete the questionnaire. Similarly, full-time and part-time students were invited to participate in the research. However, most students in our sample are full-time (94.56%). In terms of courses studied, 43.95% of students studied compulsory courses, 22.56% of students studied so-called compulsory electives (i.e., courses that are part of a compulsory block, but students choose only a certain number of courses from the available range), and 33.49% of students studied in elective courses (i.e., courses that are optional in relation to a specific course of study). In 27.80% of the cases, the course was in the form of seminars; in 40.09% of the cases, the course was mainly in the form of lectures, and in the remaining 32.10% of the cases, it was a combination of lectures and seminars.

Regarding the procedure, data collection took place over three semesters—namely, the autumn semester of 2021, the spring semester of 2022, and the autumn semester of 2022—from November 22, 2021, to December 23, 2022. First, in each of the semesters, teachers who taught their courses in an online learning environment (OLE) at the faculty were contacted and asked to include their course in the research. Each semester, we aimed to recruit at least 30 courses for which their teachers agreed to participate in the research. At the same time, these were always unique courses in each of the semesters (i.e., data were not collected from the same course in two different semesters). Then, in each of the three semesters, all students in the selected courses were approached to complete the questionnaire, which focused on various dimensions of self-regulated learning and other relevant factors. Subsequently, after the end of the semester, relevant data on students’ behavior in the online learning environment were extracted for those students who agreed to participate in the research. The entire research, i.e., the distribution of the questionnaire and the extraction of data from the selected courses in the online learning environment, was carried out in collaboration with the teachers of the selected courses and the Centre for Information Technologies, which manages the faculty’s online learning environment. The online learning environment used at the faculty was Moodle.

### Measures

#### Perceived instructional support.

To measure students’ perceived instructional support from the teacher of their course, we used a 10-item **perceived instructional support** scale used by Lee, Srinivasan, Trail, Lewis, & Lopez [48], which included items such as: “I felt that I could ask any questions regarding the course materials to the instructor”, “I felt that the instructor was easily accessible”, and “The instructor responded to students’ questions in a timely manner”. Respondents answered on a five-point Likert scale ranging from 1 (strongly disagree) to 5 (strongly agree). The Cronbach’s alpha in the original study (i.e., [48]) was 0.957; in our sample, the Cronbach’s alpha is 0.915.

#### Self-regulated learning support.

To capture how SRL is supported in OLE, we conducted a directed content analysis [49] of 11 courses purposefully selected to cover the widest possible variety of a course design within one faculty. In these courses, we aimed to code all units (tools, structures, and discourse) that might serve as SRL support. In this step, we have indicated about 60 codes that were attributed to the three phases of SRL (preparatory, performance, reflective) and, at the same time, to the type of SRL process (metacognitive or cognitive and affective or motivational). Codes were joined into categories capturing all identified ways of SRL support. Five additional courses were analyzed to ensure data saturation at this step, which led to further elaboration of categories. Critical codes in each category were selected and reformulated to formative indicators, ensuring that all previously indicated ways of SRL support were included in the scheme. The developed tool SRL-S (Self-Regulated Learning Support) was used in a quantitative content analysis of 16 courses done by three coders (one ICT researcher, one university teaching researcher, and one educational ICT specialist) [50]. After four rounds of clarification and cyclical reformulation of indicators, researchers achieved agreement on the final formulations of indicators and on coding particular features.

SRL-S consists of 55 items indicating the presence or absence of a particular SRL support process. For each item, coders decide between 1 (presence) and 0 (absence) values. Items are divided into three phases: preparatory, performance, and reflective. In each of these phases, support of two groups of processes is measured: cognitive and metacognitive processes and affective and motivational processes. Support for each process within these groups is indicated by five items. Thus, each SRL process—such as goal setting, time management, or help-seeking—is scored on a 0–5 scale indicating the degree of support provided, with higher values reflecting stronger support. The tool also produces aggregated scores across learning phases (preparatory, performance, reflective) and self-regulated learning (SRL) dimensions (cognitive/metacognitive vs. affective/motivational), calculated as the mean of the relevant process scores (thereby maintaining the original 0–5 scale). An overall SRL support score is computed as the mean of all process scores. The table below (Table 1) presents a complete version of the SLR-S coding scheme.

**Table 1:** Description of the coding scheme.

Phase of SRL	SRL dimension	SRL process supported	Items
**Preparatory phase**	Cognitive and metacognitive dimension	Goals setting	• At the beginning of the course, the content is explained (e.g., basics of Italian at A1 level; introduction to linguistics topics with a focus on German). • At the beginning of the course, the organization is explained (e.g., lecture followed by a test; required reading and an assignment in each topic). • At the beginning of the course, instructions are given on how students should work with the course (e.g., review materials, complete tests, use self-study resources, practice in seminars). • At the beginning of the course, the intended learning outcomes are stated in student-oriented terms (e.g., “by the end of the course, students will be able to identify different genres of comics”). • At the beginning of the course, the relationship to other components of the subject is declared (e.g., lectures, seminars, creative work) or it is stated that the course is delivered fully online.
		Planning, organizing	• Based on the structure of the course, teacher’s comments, or the dates attached to sections, it can be inferred that the topics were released gradually or assigned to specific time periods. There are approximately 10–12 topics, which likely correspond to a semester’s teaching. • At the beginning of the course, the teacher provides at least some indication of the expected workload (e.g., “each week you will need to complete an assignment by Wednesday”/ “in addition to the lecture you will read one chapter”). • The time requirements for individual topics are stated, or the weekly workload is approximately equivalent (e.g., each topic corresponds to one lesson, with a comparable number and size of materials). • At the beginning, the assessment methods are clearly described (e.g., “final written exam,” “continuous assessment with points”). • From the start, all deadlines are communicated (e.g., dates of midterm tests, submission deadlines), or it is specified that tasks will be due weekly.
	Affective and motivational dimension	Self-efficacy/self-esteem	• Communication from the teacher to students throughout the course is polite (e.g., appropriate forms of address, use of “please”). • Communication from the teacher to students is collegial/partner-like, encouraging participation (e.g., explicitly inviting questions, suggestions, or opportunities to influence the course content). • Communication with students is friendly across the course (e.g., greetings such as “dear students,” use of friendly emoticons, etc.). • On the course homepage, the teacher expresses confidence that students will be able to complete the course successfully if they make the effort. • At the beginning of the course, the teacher states that in exceptional cases it is possible to arrange individual agreements regarding course requirements.
		Task value (supporting intrinsic orientation)	• From the course introduction, it is clear why the course is important for students’ development or for the field of study. • The content of the course involves not only the acquisition of knowledge, but also its application or the practice of skills. • The teacher explains the logic and connections in the organization of individual topics (so that the student perceives a sense of progression from one point to another). • When presenting the conditions for completing the course, the teacher also explains their meaningfulness (e.g., why specific aspects are assessed, why students are required to complete particular tasks). • The teacher presents the subject matter and the course as interesting (e.g., by illustrating topics with historical artifacts, documentary films, works of fiction or essays, TED talks, etc.).
**Performance phase**	Cognitive and metacognitive dimension	Time management	• At some point, the teacher verbally supports students’ time management (e.g., reminding them of deadlines or assignments in forums or within a topic, highlighting what should already be studied at a given point in the semester, etc.). • The course workload is distributed regularly over time. • The majority of materials are organized by individual topics, rather than presented as a cumulative collection for the entire course. • Work for most topics is scheduled with deadlines (e.g., assignment submission dates). • Completion of topics is almost always verified through mandatory student activities.
		Help-seeking	• At the beginning of the course, a clear method for students to communicate with the teacher is established (e.g., forum, email). • Students are encouraged through various means to communicate with the teacher (e.g., “don’t hesitate to write me your questions if you don’t understand something”). • Students make use of the opportunity to ask questions and communicate with the teacher. • Ways for students to contact each other are set up (and used at least once). • The course supports peer-to-peer interaction (the teacher uses any tool that allows students to perceive the activity of others).
		Environmental structuring	• Materials are explicitly categorized as required or recommended (e.g., it is clear for each item whether it must be studied or not). • Materials are accompanied by comments explaining their role in the course (e.g., what the material is for, why it is included, when it should be studied). • The course structure is clear and well-organized. • The course does not contain an unmanageable amount of materials. • All required materials are accessible through the online learning environment, or via functional links within it.
	Affective and motivational dimension	Effort regulation	• For most topics, the teacher verbally motivates students to engage with the course. • For most topics, it is clear how students should study and what they are expected to achieve. • The teacher motivates through the interest of the study materials (e.g., videos, non-textbook readings, topic updates). • The teacher motivates by using online activation tools (quizzes and online apps for collaborative tasks). • The teacher motivates by providing opportunities for student self-expression and creativity (e.g., creative assignments, discussion posts).
		Test anxiety	• Mock tests or sample test questions are available. Alternatively, the course is completed with a seminar project or open-book tests without time limits. • Study materials include questions and content on which students should focus and which they should master after studying. • The final grade consists of multiple (at least three) components. • Activities contributing to the final grade are verbally presented by the teacher in a way that reduces anxiety (e.g., “you have enough time,” “don’t worry,” “only familiar topics are included,” “your best attempt counts,” etc.). • Clear criteria are provided for completing each component (e.g., points for essays, points for exams, etc.).
**Reflective phase**	Cognitive and metacognitive dimension	Self-evaluation	• The course provides ongoing feedback on students’ mastery of the content. • The course allows students to compare their work with peers or provides examples of student work. • Students have access to continuous information on their progress in the course. • The teacher occasionally—but obligatorily—requires students to reflect on what they have learned in a topic. • The teacher provides students with at least one instance of individualized formative feedback on their work during the course.
	Affective and motivational dimension	Self-satisfaction	• The teacher acknowledges students’ activity in the course. • The teacher acknowledges the value of students’ contributions/ participation in discussions. • Students have the opportunity to provide feedback on the course as a whole. • Students have the opportunity (and this opportunity is used at least once) to express their own opinions and perspectives on the topics covered. • The course is designed so that it cannot be completed without active mental engagement.

As mentioned above, each of the 76 courses was independently coded by three different raters. To assess the reliability of the coding procedure, we calculated the inter-rater reliability (IRR) using intraclass correlation coefficient (ICC). For the overall SRL support score, the ICC indicated 0.936. For the individual phases of SRL, the ICC indicated values 0.911 (Preparatory phase), 0.912 (Performance phase), and 0.887 (Reflective phase). For the two SRL dimensions, the ICC indicated values 0.915 (Cognitive and metacognitive dimension) and 0.917 (Affective and motivational dimension). Values above 0.75 are generally considered to be a good match, values above 0.9 are an excellent match [51], thus the results of the IRR analysis confirms that the coding procedure provides consistent ratings across different coders.

#### Students’ online learning behavior.

In order to measure students’ learning behavior in the online learning environment (OLE) in which they took courses, we extracted student log records from the OLE database and created three different proxy indicators describing different aspects of students’ online learning behavior in courses. The first indicator is based on the number of times a student visits a course; the second indicator measures the total time a student spends on a course in OLE. The third indicator focuses on the irregularity of student visits to a course. In all three cases, the way of creating/calculating the indicator of students’ online learning behavior is inspired by previous research in the field.

The **number of visits to a course** indicator conceptualizes a visit as a situation where a student enters a course in OLE, spends some time studying learning materials or participating in learning activities, and then leaves the course. When the student returns to the course after some time, this is conceptualized as the start of a new visit. Since the end of a visit is not explicitly recorded in the logging system, we use a so-called inactivity threshold to distinguish between individual visits. Specifically, for this study, we used an inactivity threshold of 30 minutes, so a 30-minute period of inactivity indicates the end of a visit [52].

To measure **the total time** a student spends in a course, we built on the individual visit detection process mentioned above (i.e., using the inactivity threshold of 30 minutes to detect the end of a student’s visit to the course). Then, we calculated the duration of the visit (in minutes) as the difference between the time of the first log entry within a visit and the last log entry within a visit prior to the 30-minute period of inactivity. However, this simple calculation would not consider the fact that after the last log entry in the database, there is still some time that a student spends in the course before actually leaving it (e.g., reading the last study material). Therefore, we followed the approach of other researchers [52,53] and estimated the so-called time spent on the last activity, which we then added to the time difference between the last and the first log entry within a visit. As for the estimation method, we estimated the time a student spent on the last activity within a visit as the average time spent on the other activities within the same visit. The total time spent by a student on a course is then the sum of the duration of all visits made by a student to the course during the semester while taking into account the time spent on the last activity in each student’s visits to the course.

The final indicator of students’ online learning behavior used in this study was the **irregularity of student visits** to a course. Using this indicator, we wanted to capture another aspect of students’ online learning behavior in courses, focusing on the amount or intensity of learning and how regularly or irregularly students learn in OLE. To measure the irregularity of visits in a course, we followed the approach suggested by [54,55] and calculated the irregularity of visits as the standard deviations of the time intervals between each visit made by a student in the course. To measure the irregularity of visits in a course, we followed the approach suggested by [54, cf. 55] and calculated the irregularity of visits as the standard deviations of the time intervals between each visit in the course. Thus, regular attendance at the course will lead to lower values of this variable, while irregular attendance at the course by the student will lead to higher values. All three variables related to students’ online learning behavior in courses showed a non-normal distribution, so we performed a logarithmic transformation before using these variables in the following analyses.

We planned to estimate multilevel models and calculate the intra-class correlation coefficient (ICC) for all three dependent variables. The ICCs for the three indicators of students’ online learning behavior are as follows: a number of visits = 0.533, irregularity of visits = 0.473, and total time spent = 0.479. The ICC can be interpreted as the amount of variance in the dependent variable caused by the differences between groups (in our case, between the individual courses in which students were enrolled). Thus, we can see that the amount of variance in all three indicators of student online learning behavior in courses is relatively high, suggesting that student behavior in courses is primarily determined by the (instructional) design of the courses, which further confirms the need to examine differences at the course level that subsequently lead to differences in the learning behavior of individual students.

#### Data analysis.

Multilevel modeling was used to analyze the data and answer the research questions of this study (cf. [56–58]). The main reason for using multilevel modeling was the hierarchical nature of the data analyzed (i.e., individual students nested within courses) and the fact that all three dependent variables analyzed showed quite high values of ICC (see above). This suggests that differences in students’ online learning behavior in courses are mainly due to differences between courses. This indicates the need to account for these differences between courses in our analyses using multilevel modeling.

In relation to the research questions and the conceptual diagram above (see Fig 1), we worked with several models in our analyses. To answer RQ1, RQ2, RQ4, and RQ5, we estimated simple random intercept models with predictors at both the individual (student) and group (course) levels using maximum likelihood (ML) as the estimation method. In order to address RQ3, we needed to use mediation analysis (see [59]). Therefore, we estimated all the models necessary to conduct the mediation analysis and assess a possible indirect effect of self-regulated learning support on students’ learning behavior. All preprocessing and analyses were performed using the R statistical software [60,61]. The *lme4* library [62] was used for multilevel modeling.

## Results

Before addressing the research questions, we conducted an initial exploration of the data, examining descriptive statistics and correlations among the variables. Basic descriptive data and correlations between all variables used in the analyses are presented in the table below (Table 2).

**Table 2:** Descriptive statistics and correlations for the variables used in the analysis.

	Min	Max	Mean	SD	1)	2)	3)	4)
1) Perceived instructional support	1.00	5.00	4.37	0.69	1			
2) Self-regulated learning support	0.79	4.30	2.42	0.77	0.19	1		
3) Number of visits in the course	0.00	5.19	3.31	0.93	0.16	0.4	1	
4) Total time spent in the course	0.87	7.82	5.62	0.97	0.16	0.38	0.79	1
5) Irregularity of visits in the course	0.03	3.86	1.30	0.70	−0.06	−0.31	−0.86	−0.61

### Research question 1

The first research question aimed to determine whether and to what extent support during learning is related to the learning behavior of students in courses in an online learning environment (OLE) in terms of its intensity and regularity. Specifically, we questioned to what extent the perceived instructional support and self-regulated learning support in OLE predict the number of course visits, the total time spent in a course, and the irregularity of course visits. To address this research question, three separate models were estimated in which the three aforementioned indicators of students’ online learning behavior were used as dependent variables, and two types of support served as independent variables. The estimated models are presented in Table 3.

**Table 3:** Effects of perceived instructional support and self-regulated learning support on indicators of students’ online learning behavior.

	Number of visits			Irregularity of visits			Total time spent		
	*Est.*	*SE*	*p*	*Est.*	*SE*	*p*	*Est.*	*SE*	*p*
***Fixed Effects***									
(Intercept)	2.05	0.27		2.06	0.21		3.98	0.28	
Perceived instructional support	0.04	0.04	0.324	**<**0.01	0.03	0.951	0.12	0.04	**0.004**
Self-regulated learning support	0.46	0.09	**<0.001**	−0.31	0.07	**<0.001**	0.45	0.09	**<0.001**
***Random Effects***									
Residual variance	0.41			0.28			0.50		
Intercept variance	0.33			0.19			0.32		
***Fit statistics***									
Marginal *R*^*2*^/ Conditional *R*^*2*^	0.149/ 0.526			0.106/ 0.468			0.142/ 0.472		
Deviance	1881.9			1456.8			2023.5		
AIC	1903.1			1479.4			2044.5		
N	879			841			872		

It can be seen from Table 3 that SRL support in courses in an OLE proves to be a significant predictor of all three types of students’ online learning behavior in courses. Thus, it can be said that the design of each course, in terms of how it reflects the teacher’s efforts to support students’ self-regulation, has a significant impact on how students behave and learn in the course, precisely how often they visit the course, how regularly they visit the course, and how much time they spend in total in the course during the semester. A higher level of self-regulated learning support in the course leads to a higher number of visits, less irregularity of visits (i.e., more regular visits), and a higher total amount of time spent in the course by a student. On the other hand, perceived instructional support from the teacher affects students’ online learning behavior only in terms of their total time spent on the course. So, the more students feel that the teacher supports them in the course (e.g., gives clear instructions, gives feedback, answers follow-up questions, etc.), the more time they will spend on the course overall.

### Research question 2

The second and third research questions aimed to explore in more detail the relationship between self-regulated learning support in OLE courses and the teacher’s perceived instructional support. Specifically, we were interested in whether or to what extent the level of perceived instructional support from the teacher is determined by the extent to which the course design focuses on supporting students’ self-regulated learning (RQ2). In other words, the extent to which the course design, in terms of its support for SRL, is reflected in students’ perceptions of their teacher and his/her ability to provide instructional support. In addition, we want to find out whether self-regulated learning support in courses affects students’ online learning behavior in OLE directly and indirectly through the teacher’s perceived instructional support (RQ3). Thus, the design of a course in OLE, in terms of its support for SRL, will affect students’ online learning behavior directly but also indirectly through its effect on students’ general perception of instructional support from the teacher.

The answer to the second research question is provided by Table 4, which indicates that there is indeed a statistically significant positive relationship between self-regulated learning support in courses and perceived instructional support from the teacher. That is, student perception of their teachers’ ability to provide instructional support is significantly informed by the design of their courses in OLE and the extent to which a course in OLE is designed with self-regulated learning support in mind.

**Table 4:** Effect of self-regulated learning support on perceived instructional support.

	Perceived instructional support		
	Est.	SE	p
***Fixed Effects***			
(Intercept)	3.96	0.13	
Self-regulated learning support	0.17	0.05	**0.002**
***Random Effects***			
Residual variance	0.37		
Intercept variance	0.09		
***Fit statistics***			
Marginal *R*^*2*^/ Conditional *R*^*2*^	0.034/ 0.228		
Deviance	1726.4		
AIC	1742.9		
N	881		

### Research question 3

**Fig 2:**
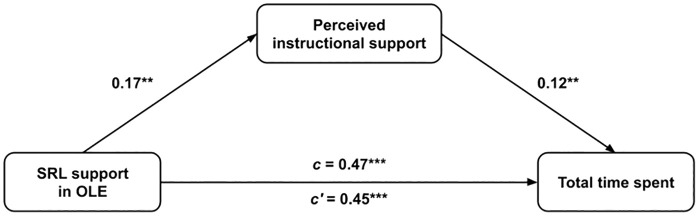
Statistical diagram of the mediation model. Here, c represents the total effect, c’ the direct effect).

To answer the third research question, we conducted a mediation analysis focusing on whether self-regulated learning support in courses indirectly affects students’ online learning behavior in OLE through perceived instructional support from the teacher. Similarly to the first research question, we carried out the media analysis three times for each indicator of students’ online learning behavior (number of course visits, total time spent in a course, and irregularity of course visits) as dependent variables (see Fig 2). The mediation analysis results show that a statistically significant indirect effect can only be detected in the case of total time spent on the course (0.021, p = 0.012). On the other hand, no significant indirect effects were found for the number (0.006, p = 0.360) and irregularity (0.001, p = 0.836) of student visits to the course. Moreover, even in the case of time spent on the course, the detected statistically significant effect can be considered rather small.

### Research question 4

Concerning the fourth and fifth research questions, we aimed to provide a more nuanced view of the effect of self-regulated learning support in OLE on students’ online learning behavior in courses by differentiating between different dimensions (RQ4) and phases (RQ5) of SRL that the support implemented in course design is aimed. Table 5 shows the resulting models in which, instead of the overall level of SRL support in the courses, a distinction was made between levels of support directed at the cognitive and metacognitive dimensions of SRL and at the affective and motivational dimensions of SRL. It is clear from the data in Table 5 that for all three indicators of students’ online learning behavior, only the effect of support focusing on the cognitive and metacognitive dimensions of SRL appears to be statistically significant, while the effect of support focusing on the affective and motivational dimensions of SRL is insignificant. Thus, support that focuses on SRL’s affective and motivational components is not directly reflected in how students behave and learn in courses in OLE.

**Table 5:** Effects of perceived instructional support and different dimensions of self-regulated learning support on indicators of students’ online learning behavior.

	Number of visits			Irregularity of visits			Total time spent		
	*Est.*	*SE*	*p*	*Est.*	*SE*	*p*	*Est.*	*SE*	*p*
***Fixed Effects***									
(Intercept)	1.77	0.28		2.29	0.22		3.72	0.29	
Perceived instructional support	0.04	0.04	0.338	0.00	0.03	0.937	0.12	0.04	**0.004**
SRL support – Cognitive and metacognitive dimension	0.64	0.15	**<0.001**	−0.49	0.12	**<0.001**	0.61	0.15	**<0.001**
SRL support – Affective and motivational dimension	−0.19	0.15	0.224	0.18	0.12	0.113	−0.17	0.15	0.266
***Random Effects***									
Residual variance	0.41			0.28			0.50		
Intercept variance	0.30			0.17			0.28		
***Fit statistics***									
Marginal *R*^*2*^/ Conditional *R*^*2*^	0.178/ 0.521			0.137/ 0.461			0.165/ 0.466		
Deviance	1875.0			1448.8			2017.3		
AIC	1900.4			1476.1			2042.5		
N	879			841			872		

### Research question 5

The last table (Table 6) then shows the models in which the individual phases of SRL (i.e., preparatory, performative, and reflective) were distinguished instead of using the overall score of SRL support. The results presented in the table suggest that it is mainly the support focused on the performative phase of SRL that significantly affects students’ online learning behavior in courses.

**Table 6:** Effects of perceived instructional support and self-regulated learning support on indicators of students’ learning behavior.

	Number of visits			Irregularity of visits			Total time spent		
	*Est.*	*SE*	*p*	*Est.*	*SE*	*p*	*Est.*	*SE*	*p*
***Fixed Effects***									
(Intercept)	2.08	0.26		2.02	0.21		4.00	0.28	
Perceived instructional support	0.04	0.04	0.303	−0.00	0.03	0.996	0.12	0.04	**0.003**
SRL support – Preparatory phase	−0.06	0.14	0.678	0.13	0.11	0.219	0.01	0.14	0.927
SRL support – Performative phase	0.42	0.19	**0.027**	−0.36	0.14	**0.011**	0.37	0.19	0.052
SRL support – Reflective phase	0.11	0.11	0.324	−0.08	0.08	0.313	0.07	0.11	0.535
***Random Effects***									
Residual variance	0.41			0.28			0.50		
Intercept variance	0.31			0.17			0.31		
***Fit statistics***									
Marginal R^2^/ Conditional R^2^	0.158/ 0.522			0.130/ 0.462			0.144/ 0.471		
Deviance	1879.0			1451.0			2022.3		
AIC	1909.0			1483.6			2052.1		
N	879			841			872		

## Discussion

The present findings provide valuable insights into the relationship between self-regulated learning (SRL) support, perceived instructional support, and students’ online learning behavior in an online learning environment (OLE). Situated within the broader context of learning analytics (LA) research, this study highlights the critical role of course design in fostering effective self-regulation and engagement in OLEs. By explicitly drawing on Zimmerman’s cyclical model of SRL [37] and the cognitive–affective distinction [47], we provide a theoretically grounded explanation of how course-level SRL support shape students’ perceptionon instructional support and their learning behavior in OLEs.

In response to RQ1, SRL support was found to significantly predict the number of course visits (β = 0.46, p < 0.001), the total time spent in a course (β = 0.45, p < 0.001), and the regularity of visits (β = −0.31, p < 0.001). Conversely, perceived instructional support from the teacher had a more limited effect, only significantly affecting the total time spent in the course (β = 0.12, p = 0.004), while showing no significant effect on the number of visits (β = 0.04, p = 0.324) or their regularity (β < 0.01, p = 0.951). This supports prior claims that structured designs embedding cognitive and metacognitive strategies promote regular engagement and persistence in OLEs [45,46,63]. At the same time, the weaker role of perceived instructional support contrasts with findings that highlight the importance of teaching presence in student engagement [64]. One explanation may be that in large-scale OLEs, design-level scaffolds are more consistently experienced by all learners, whereas individual perceptions of instructor support vary and may be less predictive of behavioral patterns. This extends earlier work [13] showing that course-level factors often outweigh individual-level variables in predicting online success.

The results addressing RQ2 confirm that SRL support significantly predicts perceived instructional support from the teacher (β = 0.17, p = 0.002). This suggests that students’ perceptions of their instructors’ support are shaped by the extent to which courses incorporate SRL-supportive elements. This aligns with the idea that learners interpret well-structured, scaffolded course designs as evidence of teacher presence and support [26]. By linking design features to perceived support, our findings extend earlier research [10,11] by empirically demonstrating that the instructional presence learners perceive is not only a function of direct interaction with the teacher but is also embedded in course design choices.

Regarding RQ3, mediation analysis showed that perceived instructional support serves as a statistically significant—but small—mediator in the relationship between SRL support and total time spent in a course (indirect effect = 0.021, p = 0.012). However, no mediation effect was detected for the number of visits (indirect effect = 0.006, p = 0.360) or the regularity of visits (indirect effect = 0.001, p = 0.836). This nuanced result refines prior discussions about the mechanisms of instructional influence. While Sitzmann and Ely [33] found mixed evidence for the role of instructional support in predicting outcomes, our study shows that instructional support’s mediating role is limited compared to the direct impact of SRL-supportive design. This suggests that, although students notice and appreciate supportive instructors, the structural presence of SRL scaffolds more directly drives engagement in OLEs. Our findings also align with previous studies that emphasize course-level interventions over instructor-driven guidance [30].

Addressing RQ4, our results indicate that SRL support focused on cognitive and metacognitive dimensions significantly predicts students’ online learning behaviors. Specifically, cognitive and metacognitive SRL support was a significant predictor of the number of visits (β = 0.64, p < 0.001), the regularity of visits (β = −0.49, p < 0.001), and the total time spent in a course (β = 0.61, p < 0.001). In contrast, SRL support aimed at affective and motivational aspects of learning was not a significant predictor for the number of visits (β = −0.19, p = 0.224), the regularity of visits (β = 0.18, p = 0.113), or the total time spent (β = −0.17, p = 0.266). This finding suggests that instructional strategies targeting goal-setting, planning, and self-monitoring (cognitive and metacognitive processes) are more effective in fostering student engagement in OLEs than those aimed at enhancing motivation alone. This aligns with research highlighting the superior predictive power of metacognitive strategies in determining students’ academic success [36,46,65]. However, it diverges from studies emphasizing the centrality of motivation, self-efficacy, and emotional regulation in OLE persistence [47,64]. A possible explanation lies in the type of outcomes measured: while affective support may influence broader outcomes such as satisfaction or course completion, it may not manifest directly in behavioral traces like login frequency or time spent in the OLE. This highlights both the explanatory power and the limitations of learning analytics in capturing the full spectrum of SRL processes, suggesting a need for multimodal data that integrates behavioral, motivational, and affective indicators.

For RQ5, our findings demonstrate that support targeting the performative phase of SRL—when students are actively engaged in learning tasks—significantly impacts engagement behaviors. Performative phase SRL support significantly predicted the number of visits (β = 0.42, p = 0.027), the regularity of visits (β = −0.36, p = 0.011), and the total time spent (β = 0.37, p = 0.052). In contrast, support focused on the preparatory (β = −0.06, p = 0.678; β = 0.13, p = 0.219; β = 0.01, p = 0.927) or reflective (β = 0.11, p = 0.324; β = −0.08, p = 0.313; β = 0.07, p = 0.535) phases showed no significant associations. This result suggests that real-time scaffolding and self-monitoring mechanisms, such as interactive prompts and immediate feedback, are crucial in sustaining student engagement. This is consistent with prior research emphasizing the importance of in-the-moment regulatory support in digital learning environments [23]. However, this is in contrast to theoretical accounts [37,42] that emphasize the cyclical interdependence of the preparatory, performance, and reflective phases. One possible explanation is methodological: behavioral traces in OLEs data capture performative regulation more directly. Preparatory and reflective processes, on the other hand, may be less visible in log data and may manifest in longer-term outcomes, such as course completion and achievement. Thus, our findings extend previous research by showing that learning analytics indicators are sensitive to processes in the performance phase. This underscores the importance of integrating complementary methods, such as self-reports and multimodal data, to capture the full SRL cycle.

Taken together, our findings converge with prior evidence emphasizing the role of metacognitive strategies and performative-phase supports in sustaining engagement, while diverging from work highlighting affective and preparatory/reflective processes as direct behavioral predictors. By systematically testing these relationships across 76 courses and integrating design-level, perceptual, and behavioral data, this study addresses long-standing concerns about generalizability in SRL–OLE research [11,14]. Furthermore, by explicitly grounding our work in Zimmerman’s cyclical model and the cognitive–affective distinction, we clarify how different dimensions and phases of SRL map onto observable engagement in OLEs, thereby advancing the theoretical integration of SRL and learning analytics.

### Limitations and future research

While the present study provides valuable insights into factors influencing engagement in Online Learning Environments (OLEs), several limitations should be acknowledged. These limitations may affect the generalizability and interpretability of the findings and should be considered in future research endeavors. One of the primary limitations of this study concerns the composition of the research sample. The study did not have complete control over who enrolled in the courses analyzed, leading to potential sampling biases. Participants may have self-selected into courses based on prior interest, experience, or motivation, which could influence engagement levels.

Additionally, variations in course difficulty, instructor effectiveness, and institutional policies were not controlled. Future studies could employ randomized controlled trials or stratified sampling techniques to ensure a more representative and comparable participant pool. Another limitation of this study is that it examined existing courses retrospectively, meaning that instructional elements were coded after course completion rather than being systematically manipulated or designed a priori. As a result, causal inferences cannot be drawn regarding the specific instructional features that enhance engagement. Furthermore, variations in course design, content delivery, and instructor involvement were not standardized across all courses analyzed. Lastly, a small portion of the data (less than 5%) was missing, primarily for the measure of irregularity of visits. It was due to low activity by some students. We addressed this issue by using pairwise deletion, which preserved most of the available data for each analysis. Due to the minimal amount of missing data, we consider this limitation to be minor and unlikely to substantially affect the results.

Future research could adopt experimental or quasi-experimental designs, allowing controlled manipulation of instructional variables to establish more apparent causal relationships, which can be used for studying unstructured data such as graphics and videos or emotional aspects of learning in OLEs [1]. Also, it should further explore the interplay between SRL support, perceived instructional support, and learning behaviors across diverse OLE settings. Given the variability in course designs and student demographics, longitudinal studies are needed to assess the long-term impact of SRL-supportive instructional strategies. Moreover, extending the current research framework by incorporating additional LA indicators—such as discussion forum interactions or assessment performance—could provide a more comprehensive understanding of how instructional designs shape online learning experiences [7]. By addressing these limitations, future studies can build a more comprehensive understanding of engagement in OLEs and contribute to developing evidence-based instructional strategies.

### Practical implications

Based on the above results, SRL-S can measure features of OLE that influence students’ behavior. Such a tool enables us to measure SRL support, contextualize data received from OLE, and thus enhance the findings of learning analytics or interventional studies. The finding that improving the level of SRL support enhances perceived teacher support and students’ learning behavior should be used to guide focused teacher support. We can quickly diagnose a particular course and scaffold teachers in supporting SRL processes that are weakly encouraged within the course. Such targeted support might be more effective than focusing on the overall development of technological competencies. SRL-S might be an easy-to-use tool for teachers’ self-evaluation or guiding teachers in designing OLE. Furthermore, a tool like SRL-S could inspire the development of a data-based framework for assessing the quality of OLE at the institutional level.

## Conclusions

In summary, this study contributes to the growing body of research on SRL and LA by empirically demonstrating the influence of course design on student engagement in OLEs. Our findings highlight the importance of embedding structured SRL support in online courses, particularly strategies targeting cognitive and metacognitive processes and the performative phase of learning. While perceived instructional support from teachers plays a role, it does not independently drive engagement as effectively as direct SRL-supportive course design. These insights underscore the need for intentional instructional design strategies to foster self-regulated learning and enhance student success in online education. From a practical perspective, our findings suggest that SRL-S is a valuable tool for measuring course features that significantly influence students’ learning behaviors. This tool enables educators to assess SRL support levels, contextualize data received from OLEs, and enhance findings from learning analytics and intervention studies. Additionally, improving SRL support enhances students’ learning behaviors and positively influences their perception of teacher support.

## Supporting information

S1 File Anonymous Research Dataset (CSV file). (ZIP)

## References

[1] Kew SN, Tasir Z. Learning analytics in online learning environment: a systematic review on the focuses and the types of student-related analytics data. Tech Know Learn. 2021;27(2):405–27. doi: 10.1007/s10758-021-09541-2

[2] Ovtšarenko O. Innovative techniques for e-learning log data processing: trends and methods. J Innov Knowl. 2025;10(5):100765.

[3] Sahni J. Is learning analytics the future of online education?: Assessing student engagement and academic performance in the online learning environment. Int J Emerg Technol Learn. 2023;18(2):33–49.

[4] Safsouf Y, Mansouri K, Poirier F. A new model of learner experience in online learning environments. In: Rocha Á, Serrhini M, editors. Information systems and technologies to support learning. Cham: Springer International Publishing. 2019. p. 29–38.

[5] Dong X, Yuan H, Xue H, Li Y, Jia L, Chen J, et al. Factors influencing college students’ self-regulated learning in online learning environment: A systematic review. Nurse Educ Today. 2024;133:106071. doi: 10.1016/j.nedt.2023.106071 38100986

[6] Osakwe I, Chen G, Fan Y, Rakovic M, Singh S, Lim L. Towards prescriptive analytics of self-regulated learning strategies: A reinforcement learning approach. Br J Educ Technol. 2024;55(4):1747–71.

[7] Schunk DH, Greene JA, editors. Handbook of Self-Regulation of Learning and Performance [Internet]. 2nd ed. Routledge; 2017 [cited 2025 Sept 1]. Available from: https://www.taylorfrancis.com/books/9781317448662

[8] Zhang Z, Costa KM, Langdon AJ, Schoenbaum G. The devilish details affecting TDRL models in dopamine research. Trends Cogn Sci. 2025;29(5):434–47. doi: 10.1016/j.tics.2025.02.001 40016003 PMC12058390

[9] Boulahmel A, Djelil F, Gilliot JM, Smits G. Towards a Skill-based Self-Regulated Learning Recommendation System. In 2023 [cited 2025 Sept 1]. Available from: https://imt-atlantique.hal.science/hal-04285013

[10] Estacio RR, Raga Jr RC. Analyzing students online learning behavior in blended courses using Moodle. Asian Assoc Open Univ J. 2017;12(1):52–68.

[11] Gašević D, Dawson S, Rogers T, Gasevic D. Learning analytics should not promote one size fits all: The effects of instructional conditions in predicting academic success. The Internet and Higher Education. 2016;28:68–84. doi: 10.1016/j.iheduc.2015.10.002

[12] Gitinabard N, Xu Y, Heckman S, Barnes T, Lynch CF. How Widely Can Prediction Models Be Generalized? Performance Prediction in Blended Courses. IEEE Trans Learning Technol. 2019;12(2):184–97. doi: 10.1109/tlt.2019.2911832

[13] Joksimović S, Gašević D, Loughin TM, Kovanović V, Hatala M. Learning at distance: Effects of interaction traces on academic achievement. Comput Educ. 2015;87:204–17.

[14] Romero C, Ventura S. Guest editorial: Special issue on early prediction and supporting of learning performance. IEEE Trans Learn Technol. 2019;12(2):145–7.

[15] Baker RS, Siemens G. Learning analytics and educational data mining. 2014.

[16] Cristea T, Snijders C, Matzat U, Kleingeld A. Unobtrusive measurement of self-regulated learning: A clickstream-based multi-dimensional scale. Educ Inf Technol. 2024;29(11):13465–94.

[17] Jovanović J, Saqr M, Joksimović S, Gašević D. Students matter the most in learning analytics: The effects of internal and instructional conditions in predicting academic success. Comput Educ. 2021;172:104251.

[18] Gašević D, Dawson S, Siemens G. Let’s not forget: learning analytics are about learning. TechTrends. 2015;59(1):64–71.

[19] Viberg O, Hatakka M, Bälter O, Mavroudi A. The current landscape of learning analytics in higher education. Comput Hum Behav. 2018;89:98–110.

[20] Roll I, Winne PH. Understanding, evaluating, and supporting self-regulated learning using learning analytics. J Learn Anal. 2015;2(1):7–12.

[21] Tempelaar DT, Rienties B, Giesbers B. In search for the most informative data for feedback generation: Learning analytics in a data-rich context. Comput Hum Behav. 2015;47:157–67.

[22] Bernacki ML, Greene JA, Crompton H. Mobile technology, learning, and achievement: Advances in understanding and measuring the role of mobile technology in education. Contemp Educ Psychol. 2020;60:101827.

[23] Dörrenbächer L, Perels F. More is more? Evaluation of interventions to foster self-regulated learning in college. Int J Educ Res. 2016;78:50–65.

[24] Azevedo R, Aleven V, editors. International Handbook of Metacognition and Learning Technologies [Internet]. New York, NY: Springer, Springer International Handbooks of Education; 2013. Available from: https://link.springer.com/10.1007/978-1-4419-5546-3

[25] Raković M, Bannert M, Molenaar I, Winne PH, Gašević D. In Conversation: Bannert, Molenaar, & Winne – Multiple Perspectives on Researching and Supporting Self-Regulated Learning via Analytics. In: Bartimote K, Howard SK, Gašević D, editors. Theory Informing and Arising from Learning Analytics [Internet]. Cham: Springer Nature Switzerland; 2024 [cited 2025 Sept 1]. p. 57–69. Available from: https://link.springer.com/10.1007/978-3-031-60571-0_4

[26] Hadwin A, Järvelä S, Miller M. Self-regulation, co-regulation, and shared regulation in collaborative learning environments. Handbook of self-regulation of learning and performance. 2nd ed. Routledge. 2017.

[27] Jansen RS, van Leeuwen A, Janssen J, Conijn R, Kester L. Supporting learners’ self-regulated learning in Massive Open Online Courses. Comput Educ. 2020;146:103771.

[28] Branch RM. Instructional Design: The ADDIE Approach [Internet]. Boston, MA: Springer US; 2009 [cited 2025 Sept 1]. Available from: http://link.springer.com/10.1007/978-0-387-09506-6

[29] Hattie J, Timperley H. The power of feedback. Rev Educ Res. 2007;77(1):81–112.

[30] Richardson JC, Maeda Y, Swan K. Adding a web-based perspective to the self-assessment of knowledge: compelling reasons to utilize affective measures of learning. AMLE. 2010;9(2):329–34. doi: 10.5465/amle.9.2.zqr329

[31] Greene JA, Azevedo R. The measurement of learners’ self-regulated cognitive and metacognitive processes while using computer-based learning environments. Educ Psychol. 2010;45(4):203–9.

[32] Juhaňák L, Juřík V, Dostálová N, Juříková Z. Exploring the effects of metacognitive prompts on learning outcomes: An experimental study in higher education. Australas J Educ Technol. 2025.

[33] Sitzmann T, Ely K. A meta-analysis of self-regulated learning in work-related training and educational attainment: what we know and where we need to go. Psychol Bull. 2011;137(3):421–42. doi: 10.1037/a0022777 21401218

[34] Gijbels D, Donche V, Richardson JTE, Vermunt JD. Learning patterns in higher education: dimensions and research perspectives. London: Routledge. 2014.

[35] Vermunt JD, Donche V. A learning patterns perspective on student learning in higher education: state of the art and moving forward. Educ Psychol Rev. 2017;29(2):269–99.

[36] Boekaerts M, Pintrich PR, Zeidner M. Handbook of Self-Regulation. Elsevier. 2000.

[37] Zimmerman BJ. Attaining self-regulation: A social cognitive perspective. Handbook of Self-Regulation. Elsevier. 2000. p. 13–39.

[38] Zimmerman BJ, Schunk DH. Self-Regulated Learning and Performance: An Introduction and an Overview. Handbook of Self-Regulation of Learning and Performance. Routledge. 2011.

[39] Pintrich PR. The Role of Goal Orientation in Self-Regulated Learning. In: Boekaerts M, Pintrich PR, Zeidner M, editors. Handbook of Self-Regulation [Internet]. San Diego: Academic Press; 2000 [cited 2025 Sept 1]. p. 451–502. Available from: https://www.sciencedirect.com/science/article/pii/B9780121098902500433

[40] Azevedo R. Theoretical, conceptual, methodological, and instructional issues in research on metacognition and self-regulated learning: A discussion. Metacogn Learn. 2009;4(1):87–95.

[41] Butler DL, Winne PH. Feedback and Self-Regulated Learning: A Theoretical Synthesis. Review of Educational Research. 1995;65(3):245. doi: 10.2307/1170684

[42] Panadero E. A Review of Self-regulated Learning: Six Models and Four Directions for Research. Front Psychol. 2017;8:422. doi: 10.3389/fpsyg.2017.00422 28503157 PMC5408091

[43] Winne PH. Construct and consequential validity for learning analytics based on trace data. Comput Hum Behav. 2020;112:106457.

[44] Winne PH. Cognition, Metacognition, and Self-Regulated Learning. In: Oxford Research Encyclopedia of Education [Internet]. Oxford University Press; 2021 [cited 2025 Sept 1]. Available from: https://oxfordre.com/education/view/10.1093/acrefore/9780190264093.001.0001/acrefore-9780190264093-e-1528

[45] Winne PH, Hadwin AF. Studying as self-regulated learning. Metacognition in educational theory and practice. Erlbaum. 1998. p. 277–304.

[46] Broadbent J, Poon WL. Self-regulated learning strategies & academic achievement in online higher education learning environments: A systematic review. Internet Higher Education. 2015;27:1–13. doi: 10.1016/j.iheduc.2015.04.007

[47] Azevedo R, Behnagh RF, Duffy M, Harley JM, Trevors G. Metacognition and Self-Regulated Learning in Student-Centered Learning Environments. In: Theoretical Foundations of Learning Environments. 2nd ed. Routledge; 2012.

[48] Lee SJ, Srinivasan S, Trail T, Lewis D, Lopez S. Examining the relationship among student perception of support, course satisfaction, and learning outcomes in online learning. Internet and Higher Education. 2011;14(3):158–63. doi: 10.1016/j.iheduc.2011.04.001

[49] Hsieh H-F, Shannon SE. Three approaches to qualitative content analysis. Qual Health Res. 2005;15(9):1277–88. doi: 10.1177/1049732305276687 16204405

[50] Boelens R, De Wever B, Voet M. Four key challenges to the design of blended learning: A systematic literature review. Educ Res Rev. 2017;22:1–18.

[51] Koo TK, Li MY. A Guideline of Selecting and Reporting Intraclass Correlation Coefficients for Reliability Research. J Chiropr Med. 2016;15(2):155–63. doi: 10.1016/j.jcm.2016.02.012 27330520 PMC4913118

[52] Kovanović V, Gašević D, Joksimović S, Hatala M, Adesope O. Analytics of communities of inquiry: Effects of learning technology use on cognitive presence in asynchronous online discussions. Internet and Higher Education. 2015;27:74–89. doi: 10.1016/j.iheduc.2015.06.002

[53] Kim D, Park Y, Yoon M, Jo I-H. Toward evidence-based learning analytics: Using proxy variables to improve asynchronous online discussion environments. The Internet and Higher Education. 2016;30:30–43. doi: 10.1016/j.iheduc.2016.03.002

[54] Jo IH, Kim D, Yoon M. Constructing proxy variables to measure adult learners’ time management strategies in LMS. J Educ Technol Soc. 2015;18(3):214–25.

[55] Kim D, Yoon M, Jo IH, Branch RM. Learning analytics to support self-regulated learning in asynchronous online courses: A case study at a women’s university in South Korea. Comput Educ. 2018;127:233–51.

[56] Heck RH, Thomas SL. An introduction to multilevel modeling techniques: MLM and SEM approaches using Mplus, third edition. Routledge. 2015.

[57] Hox J, Moerbeek M, Schoot R van de. Multilevel Analysis: Techniques and Applications. 3rd ed. New York: Routledge. 2010.

[58] Snijders TAB, Bosker R. Multilevel Analysis: An Introduction to Basic and Advanced Multilevel Modeling. 2011.

[59] Hayes AF. Introduction to mediation, moderation, and conditional process analysis, second edition: A regression-based approach. Guilford Publications. 2017.

[60] Posit team. RStudio: Integrated development environment for R. [Internet]. Posit Software, PBC; 2023. Available from: http://www.posit.co/

[61] R Core Team. A language and environment for statistical computing. [Internet]. Vienna, Austria: R Foundation for Statistical Computing; 2025. Available from: http://www.R-project.org/

[62] Bates D, Mächler M, Bolker B, Walker S. Fitting linear mixed-effects models using lme4. J Stat Softw. 2015;67:1–48.

[63] Zimmerman BJ. Becoming a Self-Regulated Learner: An Overview. Theory Into Practice. 2002;41(2):64–70. doi: 10.1207/s15430421tip4102_2

[64] Richardson JC, Maeda Y, Lv J, Caskurlu S. Social presence in relation to students’ satisfaction and learning in the online environment: A meta-analysis. Comput Hum Behav. 2017;71:402–17.

[65] Schunk DH, Zimmerman B. Handbook of Self-Regulation of Learning and Performance. Taylor & Francis. 2011.

